# Remarks on the article of Hadas et al.: Transmission of chimeric HIV by mating in conventional mice: prevention by pre-exposure antiretroviral therapy and reduced susceptibility during estrus

**DOI:** 10.1242/dmm.014043

**Published:** 2014-02

**Authors:** Deborah J. Anderson, Joseph A. Politch

**Affiliations:** Department of Obstetrics and Gynecology, Boston University School of Medicine, Boston, MA 02215, USA

We read with interest the recent paper by Hadas et al., titled “Transmission of chimeric HIV by mating in conventional mice: prevention by pre-exposure antiretroviral therapy and reduced susceptibility during estrus”, first published online on July 25, 2013 (ahead of print) in *Disease Models & Mechanisms* (Hadas et al., 2013). The paper describes an innovative HIV transmission model using conventional mice and the EcoHIV virus [HIV genome with envelope from ecotropic murine leukemia virus (MLV)]. Such a model could offer distinct advantages, as described by the authors: (1) “[this model] provides a simple, small animal platform to investigate interventions to prevent the most frequent route of HIV transmission” and (2) it opens “the extensive repertoire of genetically engineered and mutant mice to study the requirements for [HIV sexual transmission]”. Current models used for studies of HIV sexual transmission have serious limitations: macaque and humanized mouse models are very expensive, limited in availability and might not accurately reflect human HIV transmission events; *in vitro* studies using human tissue explants are limited by poor cell viability and inter-patient variability; and *in vitro* models based on human cell lines usually do not reflect the complexity of natural differentiated genital tract epithelia. An inexpensive murine model for HIV transmission studies would be a welcome addition to the field, but, unfortunately, the new model described in this article also has serious limitations. We cite three major limitations here:

1. The paper showed that infectious virus was present in reproductive tract tissue (vas deferens) of EcoHIV-infected male mice, but did not demonstrate its presence in semen, nor did it establish that the virus acquired by females co-housed with infected males was sexually transmitted via semen. Transmission could have occurred through biting or exposure to urine or feces. Male mice, including those of the C57BL/6J strain used in this study, are aggressive and have been shown to bite females throughout the estrous cycle (Canastar and Maxson, 2003), as well as following copulation (McGill, 1962). Furthermore, female-to-male and intra-female aggression are not uncommon in mice (Brain, 1999; Morè, 2008) and could facilitate the transmission of virus among group-housed animals. Indeed, a pioneering study on ecotropic MLV (Portis et al., 1987) showed that saliva from infected mice contained high concentrations of infectious virus and that, at least among males, the virus was probably transmitted through biting.
2. Even if the virus was transmitted via semen, the mechanisms of transmission would be different in mice than humans. One difference is the insemination site. Human semen is deposited in the vagina, whereas, in mice and other rodents, most of the semen enters the uterine cavity (Sobrero and MacLeod, 1962; Zamboni, 1972). Furthermore, the mouse penis, unlike the human penis, has spines (Rodriguez et al., 2011), which could cause vaginal abrasions during copulation. Finally, the receptor for the ecotropic MLV is a cationic amino acid transporter (CAT-1) that is ubiquitously expressed on almost all murine cells (Hatzoglou et al., 2004), whereas the primary HIV receptor, CD4, and its co-receptors, CCR5 and CXCR4, are expressed primarily on immune cells.
3. The experiment in this paper that proportedly demonstrated reduced virus susceptibility during estrus was poorly designed. The infection rate of females caged with infected males for one night during estrus was compared with the infection rate of nonsynchronized females caged with infected males for several days. This study can only be meaningful if the infection rate per number of exposures is calculated during each specific phase of the estrous cycle, and other potential routes of exposure are eliminated. A proper experiment studying viral transmission by intercourse on different days of the estrus cycle would be difficult to execute because female mice preferentially mate during estrus, and do not mate at all during diestrus (Fowler and Edwards, 1957).

In our opinion, this article has not established a new mouse model for HIV sexual transmission and, even if subsequent experiments show that the EcoHIV virus can be transmitted via intercourse in conventional mice, the utility of the model for studies on mechanisms of human HIV sexual transmission is severely limited due to differences in viral characteristics between EcoHIV and HIV, and in the events associated with intercourse and reproduction in mice and humans.

## References

[b1-0070177] Brain P. F. (1999). Aggression in female mammals: is it really rare? Behav. Brain Sci. 22, 218

[b2-0070177] Canastar A. Maxson S. C. (2003). Sexual aggression in mice: effects of male strain and of female estrous state. Behav. Genet. 33, 521–528 14574129 10.1023/a:1025722700138

[b3-0070177] Fowler R. E. Edwards R. G. (1957). Induction of superovulation and pregnancy in mature mice by gonadotrophins. J. Endocrinol. 15, 374–384 13475597 10.1677/joe.0.0150374

[b4-0070177] Hadas E. Chao W. He H. Saini M. Daley E. Saifuddin M. Bentsman G. Ganz E. Volsky D. J. Potash M. J. (2013). Transmission of chimeric HIV by mating in conventional mice: prevention by pre-exposure antiretroviral therapy and reduced susceptibility during estrus. Dis. Model. Mech. 6, 1292–1298 23886803 10.1242/dmm.012617 PMC3759349

[b5-0070177] Hatzoglou M. Fernandez J. Yaman I. Closs E. (2004). Regulation of cationic amino acid transport: the story of the CAT-1 transporter. Annu. Rev. Nutr. 24, 377–399 15459982 10.1146/annurev.nutr.23.011702.073120

[b6-0070177] McGill T. E. (1962). Sexual behavior in three inbred strains of mice. Behaviour 19, 341–350

[b7-0070177] Morè L. (2008). Intra-female aggression in the mouse (Mus musculus domesticus) is linked to the estrous cycle regularity but not to ovulation. Aggress. Behav. 34, 46–50 17647257 10.1002/ab.20217

[b8-0070177] Portis J. L. McAtee F. J. Hayes S. F. (1987). Horizontal transmission of murine retroviruses. J. Virol. 61, 1037–1044 3029398 10.1128/jvi.61.4.1037-1044.1987 PMC254060

[b9-0070177] Rodriguez E. Jr Weiss D. A. Yang J. H. Menshenina J. Ferretti M. Cunha T. J. Barcellos D. Chan L. Y. Risbridger G. Cunha G. R. (2011). New insights on the morphology of adult mouse penis. Biol. Reprod. 85, 1216–1221 21918128 10.1095/biolreprod.111.091504 PMC3223253

[b10-0070177] Sobrero A. J. MacLeod J. (1962). The immediate postcoital test. Fertil. Steril. 13, 184–189 13914711 10.1016/s0015-0282(16)34447-8

[b11-0070177] Zamboni L. (1972) Fertilization in the mouse. In Biology of Mammalian Fertilization and Implantation (ed. Moghissi K. S. Hafez E. S. E.), pp. 213–262 Springfield, IL: Charles C. Thomas

